# Experimental Evidence on the Possible Use of Fine Concrete and Brick Recycled Aggregates for 3D Printed Cement-Based Mixtures

**DOI:** 10.3390/ma18030583

**Published:** 2025-01-27

**Authors:** Marco Pepe, Rosario Lombardi, Carmine Lima, Bruno Paolillo, Enzo Martinelli

**Affiliations:** Department of Civil Engineering, University of Salerno, 84084 Fisciano, Italy; mapepe@unisa.it (M.P.); lombarosar@gmail.com (R.L.); clima@unisa.it (C.L.); brpaolillo@unisa.it (B.P.)

**Keywords:** 3D printed concrete, construction and demolition waste, recycled concrete, experimental methods, 3D printed concrete rheology, 3D printed concrete mechanical properties

## Abstract

In recent years, the development of alternative and more sustainable technologies for reinforced concrete structures has been attracting more and more interest, given the increasing need to reduce the impact that the construction sector has on the environment. Furthermore, 3D concrete printing (3DCP) technology falls into this context, allowing the optimization of the quantities of employed raw material to be used while at the same time allowing the possibility to design more complex elements’ shapes. In the view of improving the sustainability of construction sector, the present study aims at experimentally investigating the characteristics of the fresh and hardened states of concrete mixtures incorporating different percentages of replacement of the fine aggregate with recycled aggregates of different nature. As such, the key innovative aspect of the present study is the possible investigation of cement-based mixtures produced with 100% recycled fine aggregates (both derived from concrete waste and brick elements) without affecting either the fresh or hardened mechanical properties of the resulting Recycled Aggregate Concrete (RAC) mixtures. Furthermore, in order to make this study linked to 3D printing technology, extruded concrete elements were realized and tested through a process designed to simulate the automated 3D concrete printing process: in fact, the RAC mixtures were designed in order to obtain an adequate workability and compressive strength typically adopted for ordinary 3D printed mixtures. Although some adjustments and further analyses are required in order to optimize the shape retention and stability, as well as the well-known problem of the 3D mixtures being linked to anisotropic behavior, the obtained results unveil that it was possible to observe promising characteristics for the mixes containing recycled aggregates (i.e., consistency index at the fresh state above 150 mm and compressive strength at 28 days above 50 MPa), which were in any case suitable for the creation of 3D printed structural concrete elements and can be further confirmed with future studies in order to validate their possible buildability.

## 1. Introduction

Sustainability has gained more and more relevance in the construction sector. The need to reduce the sector’s environmental impact has led to a search for alternative solutions that are capable of satisfying the requests that modern problems make necessary and urgent. More specifically, emphasis has been placed on reducing the use of concrete and, therefore, of cement, which is the constituent of concrete whose production is characterized by the highest energy demand and, thus, environmental impact. In this context, digital fabrication techniques with concrete have taken hold as a promising solution, as they can lead to reducing the quantity of materials need to realize a given structural member [1,2,3,4].

The present study refers to 3D printing of concrete (3DPC), also known as additive manufacturing (AM), which is a set of techniques and technologies that is based on forming complex shapes from digital models, involving the superposition of multiple layers of material [4,5]. This technology has had strong development in recent years [6], with applications to structural members [7].

The possible use of 3DPC has recently been explored for structural systems as diverse as arch structures, segmental beams, and lightweight slabs, where dedicated digital tools have been used with the aim to optimize their shapes [8]. However, real-world realizations of full-scale houses and buildings are more and more common [9].

In the production of 3DPC, the material properties at both the fresh state and hardened state are of relevance, as they can affect the ability to shape 3DPC members and provide them with the mechanical properties requested for structural applications [2,5,10].

In relation to fresh concrete, it is possible to define some parameters that indicate whether the reference mix has the appropriate characteristics to be printed out. The most relevant ones are listed below [10]:

- Pumpability, i.e., the ability of the mix to pass through the pressurized pump;
- Extrudability, i.e., the ability of the mix to come out of the nozzle of the printing machine in a manner adequate for printing itself (continuous and constant layer, no segregation);
- Shape retention, i.e., the ability of the mortar to maintain the shape obtained once extruded from the nozzle;
- Printing open time, i.e., the time interval associated with each mix within which the physical and mechanical characteristics of the mixture are such as to allow it to be printed;
- Buildability, defined as the ability of the mix to be printed in multiple layers up to a pre-established height without these collapsing on themselves.

Once hardened, it is necessary to define the mechanical behavior of the 3DPC elements, which obviously do not have the same characteristics as those made of conventional concrete. In fact, the studies conducted so far have pointed out that the realization in layers leads to an anisotropic behavior for 3DPC elements, depending on the direction of application of the load. More specifically, the layered printing process leads to a layered structure whose responses depend, on the one hand, on the properties of the single layers and, on the other hand, on the bond between the layers [11,12,13,14,15,16].

Another relevant aspect that makes the use of 3DPC technology increasingly interesting is the replacement of all or part of the elements constituting the mortar mix itself with materials that have similar characteristics but are characterized by a lower environmental impact, in order to make it more sustainable [17,18,19,20,21]. In this sense, a generally used procedure that also concerns the following study is to replace ordinary aggregates with recycled ones obtained from the demolition of existing buildings (so-called Construction Demolition Waste, or CDW) [22]. Obviously, the use of such materials also leads to a series of problems to take into account, mainly related to the variability in the characteristics of the latter (these often come from different sources); for example, a decrease in the durability of 3DPC elements, difficulty in formulating easily reproducible and standardized mixtures, a higher porosity of the elements, and a possible decrease in flexural compressive strength [10,23].

Starting from what was described above, the present study aims at analyzing the physical and mechanical characteristics of a series of different mixtures produced by replacing conventional aggregates with recycled aggregates of various types in different percentages. Furthermore, in order to understand whether recycled aggregates are actually suitable for 3DPC, some specimens were produced for the same mixtures by simulating the extrusion process which is the basis of 3DPC. For each of the specimens, the spreading values and the compressive and flexural strength values were determined. More specifically, 3DPC specimens were tested in three-point bending, which showed a substantial consistency of the observed mechanical resistance.

This paper is structured as follows. Section 2 describes the materials used for the creation of the mixes, as well as the methodologies for the creation of the 3D elements and the physical and mechanical tests performed. Section 3 presents the results obtained, as well as a series of analyses of the same. Finally, Section 4 reports the conclusions that could be deduced from the work carried out.

## 2. Materials and Methods

This section describes the composition and preparation of the mixtures under consideration. The workability of these mixtures was observed and measured, with the aim to identify which of them are suitable for the application of the 3D concrete printing (3DCP) technique. Consequently, 3DPC elements were produced using the chosen mixes; these were then subjected, together with conventionally cast prisms, to bending tests in order to evaluate their mechanical behavior.

### 2.1. Constituents

**Figure 1:**
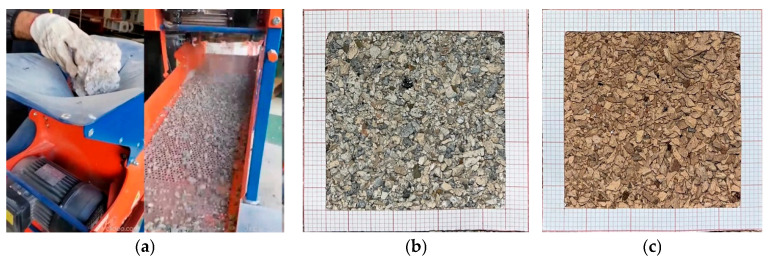
Recycled fine sand: (**a**) production process, (**b**) RCA, and (**c**) RMA.

The main constituents used for the production of mortars are the following:

- Portland Cement type CEM II 42.5 [12];
- Natural fine sand (maximum nominal diameter equal to 2 mm) characterized by a particle density equal to 2600 kg/m^3^ and water absorption capacity at 24 h equal to 2.00% [13];
- Recycled Concrete fine Aggregates—RCA (maximum nominal diameter equal to 2 mm) obtained from concrete crushing (see Figure 1) and characterized by a particle density equal to 1750 kg/m^3^ and water absorption capacity at 24 h equal to 17.60% [13];
- Recycled Masonry fine Aggregates—RMA (maximum nominal diameter equal to 2 mm) obtained from brick crushing (see Figure 1) and characterized by a particle density equal to 1870 kg/m^3^ and water absorption capacity at 24 h equal to 17.60% [13];
- A polycarboxylate ether superplasticizer commercially labelled “MasterGlenium ACE 442” [14].

### 2.2. Mixtures

The mixtures under consideration consist of the following:

- A binder, coinciding with Portland-type cement, in constant quantity between the various mixtures;
- Fine aggregates of various nature, having a maximum diameter of 2 mm;
- Water;
- A superplasticizer, necessary to improve the workability of the mixture.

The mixtures differ from each other primarily in the quantity and nature of the aggregates used; according to the latter, the quantity of water and additive necessary to reach the necessary resistance and workability is varied.

Specifically, 6 mixtures were formulated: the production started from a reference one in which only conventional aggregates were used, then replaced (by volume) with recycled aggregates in different percentages (25%, 50%, and 100%, according to the cases). The latter is of two kinds: aggregates deriving from the crushing of red clay bricks (RMA, Recycled Masonry Aggregates) and aggregates deriving from the crushing of demolished concrete elements (Recycled Concrete Aggregates). These were appropriately sieved to obtain a maximum diameter of 2 mm.

As mentioned, given the different nature of the aggregates used and, in particular, their ability to absorb the water in the mixture, this results in the variation of the amount of water used. More specifically, to determine the amount of additional water needed, a water absorption of 2% for f_NAT_ and 17.60% for f_RCA_ and f_RMA_ was considered. Obviously, these values are proportional to the quantities used in every mix.

At the same time, depending on the consistency of the mixture at the time of its creation, the value of the additive used was adjusted as specified in the following table below representing the mortars produced in this study.

**Figure 2:**
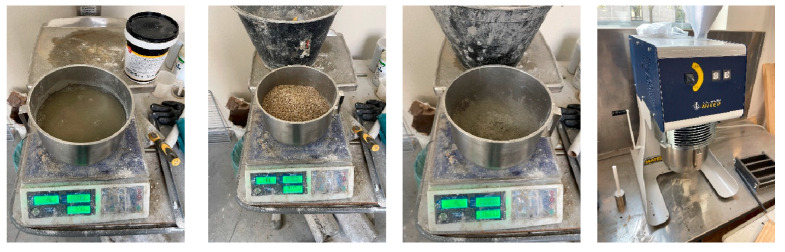
Weighing of components and mortar mix preparation.

Figure 2 shows some representative images of the mixtures’ preparation.

As expected, this process was not immediately effective, but it was necessary to carry out a series of tests to understand the correct value of additive that was required to obtain an adequate workability for 3D printing.

**Figure 3:**
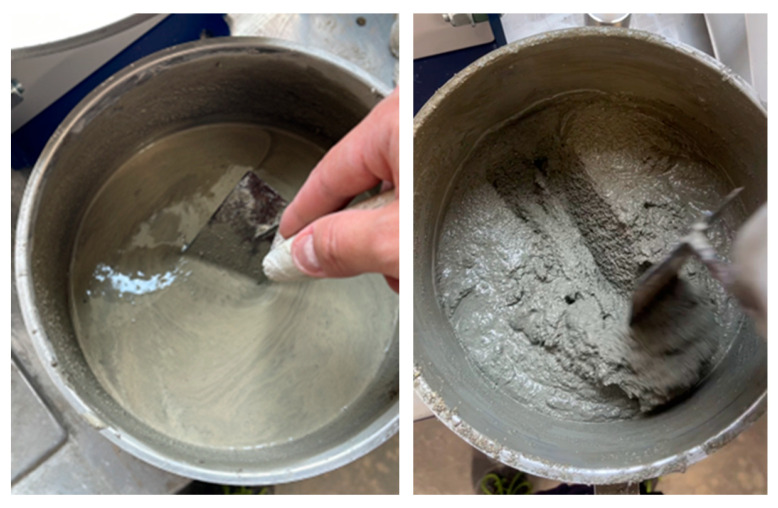
Mixtures appearance before and after adjusting the amount of additive.

This process consisted of a gradual increase in the amount of superplasticizer during the mixing phase of the mixture. Specifically, starting from the value related to the reference mix (i.e., 2.50 kg/m^3^ as also reported in Table 1), following an initial visual observation of the viscosity of the mixture, it was assessed for each mix whether the addition of further plasticizer was necessary. In these cases, the superplasticizer was progressively added to the mixture with the aim of reaching the target consistency of the mixture. Obviously, following each addition, the time necessary for the superplasticizer to have an effect on the mixture was waited (3–5 min). Once the mixture had reached—based on visual observation and following manipulation with a trowel—the target consistency, the mix was extruded as specified in the following Section 2.3. For instance, the mixtures labelled “standard” and RMA50 were made twice: in the second attempt, the overall amount of superplasticizer was reduced, as workability turn out to be too high in the first attempt. Additionally, for the RCA, the mixture composition was defined as a result of a trial and error process which led to identifying the quantity of admixture necessary to obtain a consistency suitable for the creation of the extruded elements (Figure 3).

### 2.3. Realization of 3DPC Specimens

**Figure 4:**
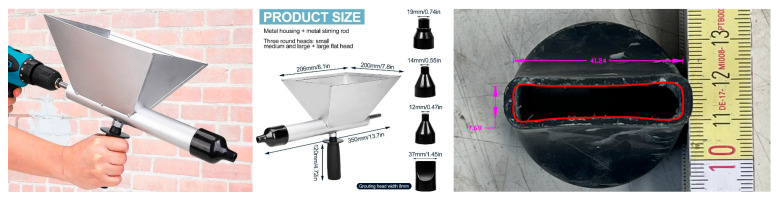
Manual hopper for mortars and its nozzle geometry [24].

As already mentioned, the resulting optimized mixtures were used to create extruded elements, thus trying to simulate the production of 3D printed concrete. The process consisted of using a manual steel hopper specifically for applying mortars, set in motion using a cordless screwdriver. Figure 4 shows the main properties of the manual device utilized in extruding the 3DPC specimens tested as part of the present study.

**Figure 5:**
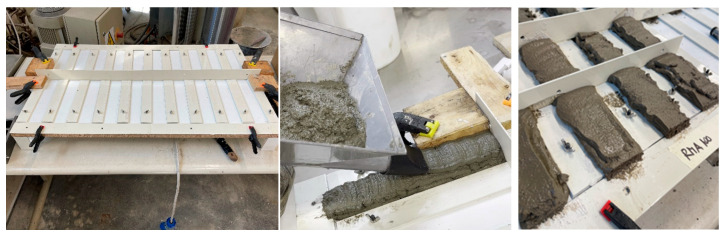
3DPC specimens production process.

The mortar was inserted into the upper housing. Among the available nozzles, the one used for the production of 3DPC had a flat form with a measured width of about 42 mm and a height of about 8 mm, with the aim of obtaining regular rectangular layers (Figure 5). The specimens were produced in a formwork having a width of 50 mm for a length of 250 mm. The latter half of the specimens were subsequently divided in half to obtain elements having a length of approximately 125 mm. The final number of specimens produced for each mixture is equal to 6. Figure 5 shows three key stages of the 3DPC specimens’ production process.

**Figure 6:**
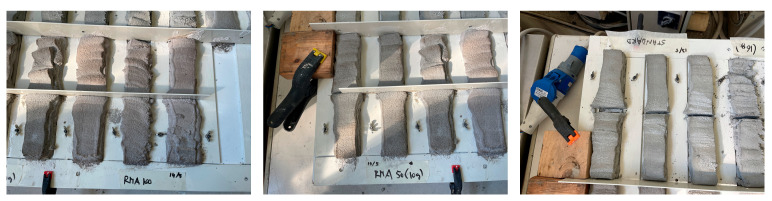
Example of 3DPC specimens after hardening.

Once hardened, these elements were removed from the formwork, wrapped in plastic film, and placed in the same environment in which the mortar prisms were left to mature, so as to avoid any variation in the results due to environmental factors (Figure 6).

### 2.4. Consistence Measurement

After the various concrete mixtures were made, the consistency of the fresh mortar was measured using the “EN 1015-3:2013—Methods of test for mortar for masonry—Part 3: Determination of consistence of fresh mortar (by flow table)” standard [25], which describes in detail the tools to be used and the procedure to be followed.

**Figure 7:**
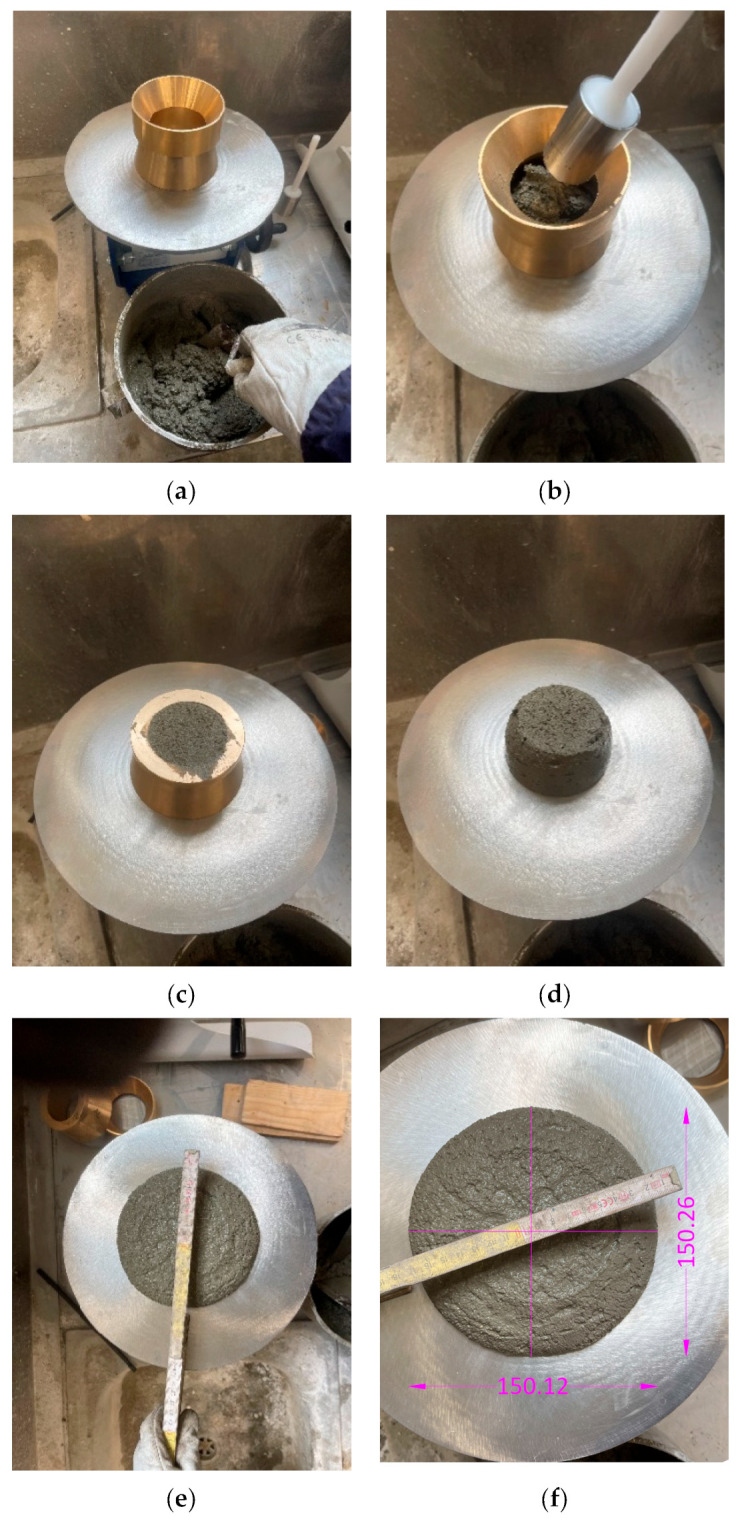
Flow table test process. (**a**) Preparing the test; (**b**) compaction of the mortar; (**c**) uniform surface at the top of the mold; (**d**) mortar after the lifting of the mold; (**e**) final result; and (**f**) measurement after scaling the photo using CAD (AutoCAD 2024) software.

Specifically, a flow table was used, consisting of a stand, a rigid table plate and disc, a horizontal shaft and lifting cam, and a lifting spindle. In addition, a stainless steel conical element, with a smooth internal surface, and a tamper, necessary to compact the concrete inserted inside the mold during the test, were provided with the latter. The dimensions of the listed instruments are indicated in the reference standard (Figure 7).

The main stages of the process are listed hereafter:

1. The first operation consisted of cleaning the circular plate on which the test was to be performed using a damp cloth and then drying it.
2. Once this was done, the mold was placed in the center of the plate and then the concrete was inserted in two successive moments, proceeding with the compaction of the same using the tamper by hitting it at least 10 times. Once this operation was completed, it was necessary to remove any excess mortar, thus obtaining a uniform surface at the top.
3. Finally, the steel mold was slowly lifted, applying 15 consecutive jolts using the flow table system itself, at a frequency of approximately 1 jolt per second. Once this was done, the diameter of the spread mortar was measured in two directions perpendicular to each other. This last operation was performed using CAD software, appropriately scaling the photo taken of the test.
4. The spread value was calculated as the average of the measured values.

### 2.5. Flexural and Compressive Strength Test on Mortar

Following the indications of the “EN 1015-11:2013—Methods of test for mortar for masonry—Part 11: Determination of flexural and compressive strength of hardened mortar” standard [26], flexural and compression tests were carried out on the mixes produced.

In particular, for each mix, prisms of dimensions 160 × 40 × 40 mm were realized. The temperature and Relative Humidity during curing were kept controlled in laboratory environmental conditions at a temperature of 20 °C and RH (Relative Humidity) between 35–40%. Once the samples had been made and left to cast for 28 days, they were subjected to a flexural strength test. After that, the elements resulting from the splitting were subjected to a compression strength test. Consequently, the flexural tests were performed on 3 specimens for each mixture, while the compression tests were performed on 6 specimens for each mixture.

**Figure 8:**
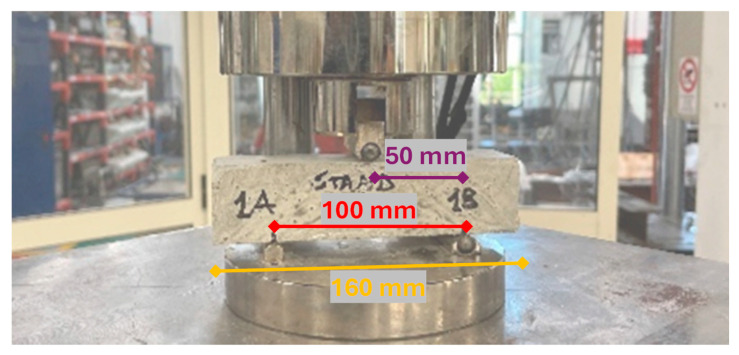
Flexural strength test configuration (after [26]).

The reference scheme for the flexural strength test is described in Figure 8.

As indicated by the standard, the load must be applied constantly, so that the break occurs within the first 90 s from the start of the test, in a value between 10 N/s and 50 N/s. The loading speed chosen for these tests was 30 N/s.

Once this was done, the maximum force reached was recorded and the flexural strength was calculated using the following relationship:

(1) $$f=1.5\cdot \frac{F\cdot \;l}{b\cdot d^{2}}$$

where

- *f* is the flexural strength;
- *F* is the maximum load applied;
- *l* is the distance between the two base supports;
- *b* and *d* are the width and the dept of the specimen.

The elements resulting from the breakage of the specimen during the flexural strength test were used to determine the compressive strength. Specifically, the test consisted of compressing the elements with a load defined by the standard as a function of the expected strength class for the mortar. In this specific case, the chosen loading speed was equal to 400 N/s. In this case too, the maximum force reached at the breakage of the specimen was recorded.

The relationship used to determine the compressive strength is as follows.

(2) $$f_{c}=\frac{F}{b\cdot d}$$

where

- *f_c_* is the compression strength of the specimen;
- *F* is the maximum load applied;
- *b* and *d* are the width and the dept of the specimen.

**Figure 9:**
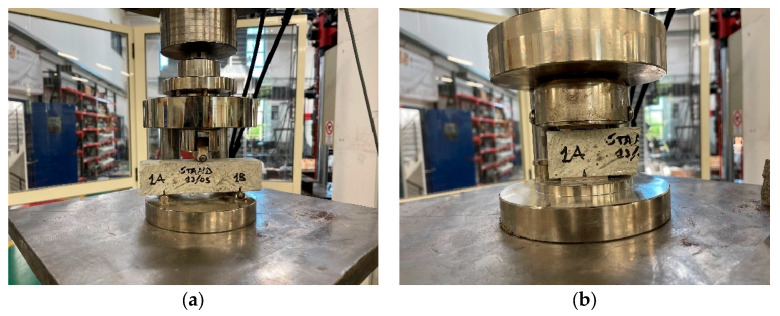
(**a**) Flexural strength and (**b**) compressive strength tests’ execution.

Figure 9 shows pictures from the bending (Figure 9a) and compressive tests (Figure 9b).

### 2.6. Flexural Strength Test on 3DPC

**Figure 10:**
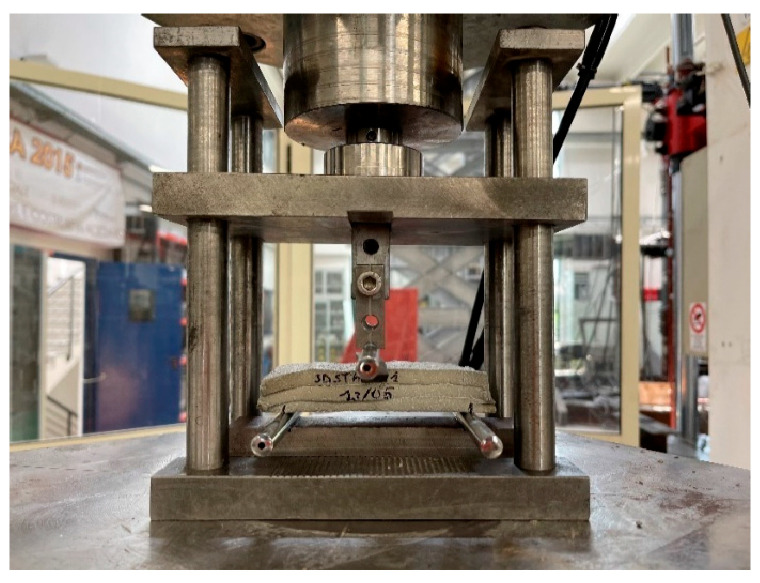
Flexural strength test setup for 3D printed specimens.

Following what is described in the EN 1015-11:2013 standard, a similar setup was created in the laboratory to test the extruded specimens. Figure 10 presents an image of the test performed.

**Figure 11:**
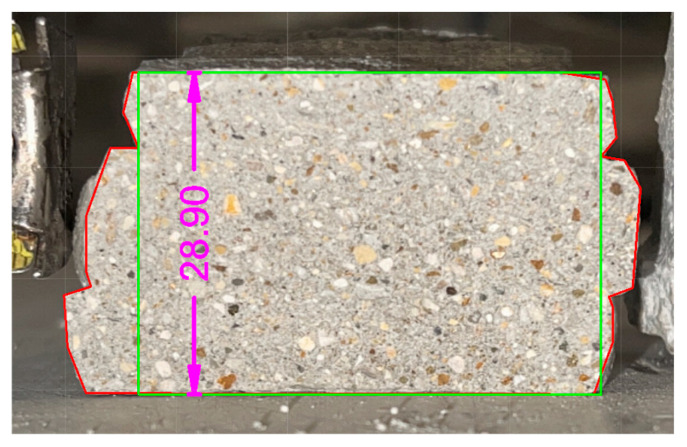
Determination of the main geometric properties of the extruded specimen.

The same relationship was used to calculate the flexural strength. Specifically, the distance between the supports was set equal to that required by the standard. As regards the dimensions of the section—*b*, *d*, and the cross section of the specimen—these were obtained from each specimen after the execution of the test itself. Here (Figure 11) is an example:

To determine the effective area, the breaking section of each specimen was observed, evaluating whether it presented discontinuity points or whether it could be considered entirely reactive during the test. Following this evaluation, the red area was determined (through CAD—AutoCAD 2024-software).

## 3. Results

### 3.1. Mixtures Characteristics

First of all, it is worthwhile to analyze the concrete’s behavior at the fresh state of the printed samples; specifically, its extrudability, shape retention, printing open time, and buildability.

**Figure 12:**
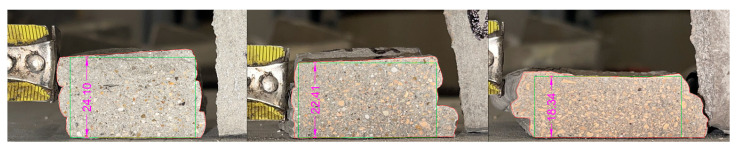
Different shapes for different flow values: REF (flow value 150.20 mm), RMA50 (flow value 180.25 mm) and RMA 100 (flow value 241.35 mm).

More specifically, the following observations can be made:

- Regarding the production of the mixtures, an interesting aspect to highlight is the rapid change observed in the workability of the mortar once mixed, in particular when recycled aggregates were used. The consequence of this was the need, on the one hand, to wait an adequate amount of time in order to understand how the workability evolved; and on the other hand, to adjust the mix also in function of this parameter, so as to increase the time in which it presented a workability adequate for the purposes of this study.
- As the amount of the superplasticizer increased, an increase in the difficulty of “pumping” the mortar was registered. As a matter of fact, this mortar was more workable but at the same time more viscous: this characteristic therefore made it more difficult for the helical element of the manual hopper to “pick up” the mortar for its extrusion;
- As the workability of the mixture increased, its ability to maintain the nozzle shape decreased. For the analyzed mixtures, this is shown in Figure 12;
- As observed before, the printing open time slightly decreased with the use of recycled aggregates. This is probably due to the water absorption capacity of the recycled particles, which reduced the workability of the mixture more quickly;
- Given the few layers made for the printed samples, it was not possible to fully analyze their buildability; that is, the ability of the mixtures to be printed for significant heights without significant deformations or collapses. However, keeping in mind what was described regarding “shape retention” in the previous points, it is possible to deduce that the workability achieved for RMA100 is too high to allow effective printing, even for higher section heights. This characteristic can be addressed by considering the possible variation of the overall amount of additive to be included within the mixture.

**Figure 13:**
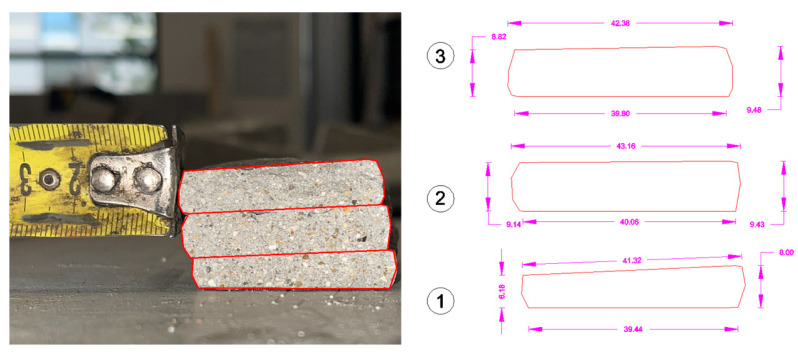
CAD extrapolation of single-layer dimensions for each 3DPC specimen (1, 2 and 3 representing the measurements for each extruded layer).

**Figure 14:**
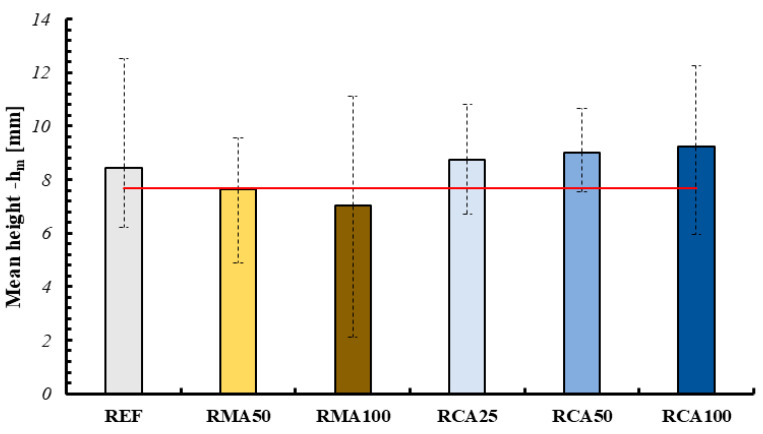
Variation of average height of layers (red line represents the height of the nozzle).

**Figure 15:**
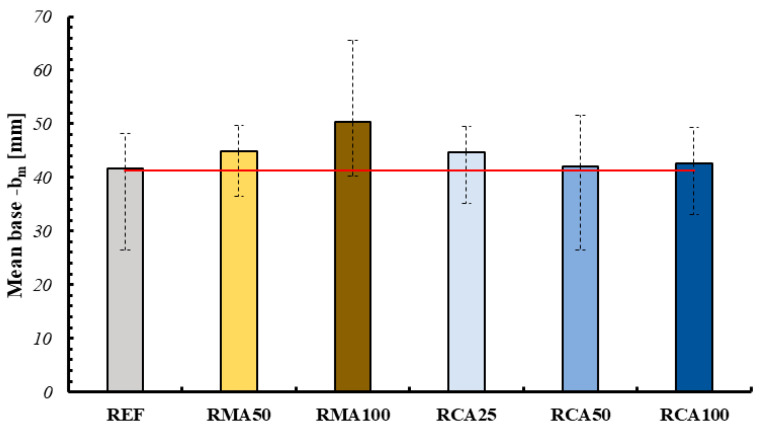
Variation of average width of layers (red line represents the width of the nozzle).

In order to determine the shape retention and stability, the base and average height of the individual layers were measured for each mixture. Specifically, the measurements were performed using CAD (AutoCAD 2024) software, extrapolating from an image of the section divided in half (after being tested under the flexural three-point bending tests), the maximum and minimum values for the parameters, and then the average value (see Figure 13). This procedure was performed for the three layers for each sample, as reported in the following figure. The key results of this analysis are presented in Figure 14 and Figure 15.

All values were compared to the nozzle dimensions, represented by the red horizontal line in the graphs. As can be observed, for the specimens containing RMA the section tends to lower and widen for the mixtures associated with higher spreading values; on the contrary, for the specimens containing RCA it is possible to observe a substantial constancy of these values.

Comparing these values with the dimensions of the nozzle, it is possible to observe, first of all, a more marked variation in the height of each layer compared to its width. Furthermore, once again for RMA it is possible to observe a greater variation, with RMA100 having an average height of the layers of about 30% less and an average base of the layers of about 12% more. On the contrary, for RCA we observe more contained and more or less constant variations with the variation of the percentage of aggregate (for example, for RCA100 we have a variation of −7.83% for the average height and −5.08% for the average base of the layers).

### 3.2. 3DPC Production Method Limitation

It is worth mentioning that the methodology used to produce the extruded and tested 3DPC elements produced in this study may not be directly comparable with the production technique realized by automated 3D mixture printers.

First of all, one of the differences concerns the way in which the mortar is “pushed” towards the nozzle: in this study, the mortar was placed inside the appropriate housing of the extruder and pushed inside the Archimedean screw manually by the operator, which involves a not high overpressure on the mortar; differently, in the case of the mortar pumps used to produce the automated 3DPC elements, this operation is completely mechanized, obviously leading to the achievement of higher pressures—due to the need to make the mortar travel a greater distance from the mixing point to the extrusion point. This aspect is important, since higher pressures correspond to the occurrence of phenomena for the mortar, such as:

- Pressure-induced phase segregation, a phenomenon in which the applied pressure is transmitted from the mixing water to the other ingredients, thus causing a separation of the same, with a loss of homogeneity linked to the fact that the water is “expelled” from the mortar (bleeding) [27].
- Shear-induced particle migration (SIPM), a phenomenon in which the difference in shear speed inside the pumping tube between the internal part and the perimeter part—due precisely to the shear that is generated between the lateral surface and the mortar—causes a migration of the particles from the outside to the inside of the tube. This creates a layer of mortar poor in aggregates at the interface and a greater concentration of the same in the central part of the pumping tube. This phenomenon, as can be imagined, is strictly linked to the speed with which the mortar is extruded, and therefore to the pressure to which it is subjected during the pumping phase [1].

This means that the produced mixtures, even if adequate for the manufacturing methodology used, require further modifications and adjustments to adapt them to use with a specific 3D printing machine.

Furthermore, there are other mortar pumping systems that can be used, which obviously pose different problems for the mortar itself that cannot be observed through the process used [28].

Finally, each layer of mortar was made manually: this involves a variability linked to the speed of making each layer, to the inclination given to the extruder itself, to the time necessary for the creation of the elements (a function first of all of the ability of the mortar to be extruded). Differently, when using a specialized machine all these parameters are controlled and constant.

### 3.3. Consistency Measurement

**Figure 16:**
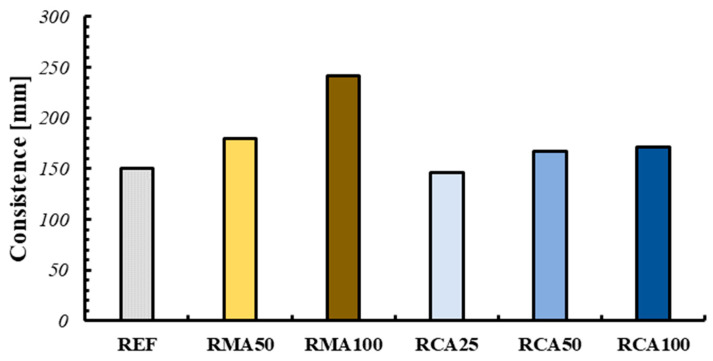
Consistency values.

The consistency measurements of the mixtures produced the results reported in Figure 16.

The values are obviously affected by the amount of superplasticizer used in each mixture. Following the creation of the extruded samples, it was possible to note how the ideal consistency for the creation of 3DPC is between 150 mm and 180 mm. For lower values, the mixture is too “dry” and therefore not suitable for extrusion. Conversely, for higher values the mixture is too fluid and at the same time too viscous, and therefore not suitable for either its extrusion or for maintaining the shape of the element.

In all cases, a high viscosity causes difficulty in extruding through the nozzle.

### 3.4. Section Area

Two alternative definitions of the section areas were considered in the present study. The first value was equal to the area of the perimeter of the specimen, including all the imperfections of the perimeter itself. The second one was equal to the area of the rectangle inscribed within the previously described perimeter. However, only the first value was considered for the calculation of the flexural strength of the 3DPC specimen. The second area was instead considered for the determination of the height *d* of the reference section (see Equation (2)).

**Figure 17:**
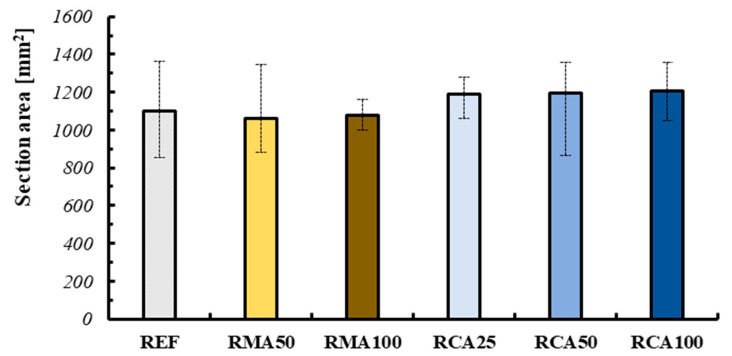
Section area.

The values of the area considered for determining the bending and compression strength are represented in the bar graph reported in Figure 17. As can be seen, the value of the area is almost invariant for all the mixtures under consideration. This is a positive aspect, since it indicates a substantial homogeneity of the samples produced, despite the differences related to the viscosity and therefore to the geometry of the final cross sections.

Furthermore, the error affecting the measure of section areas for each mixture can be considered as a yardstick for evaluating the quality of a mix with regard to 3D printing. As a matter of fact, a lower variability of the cross-section can indicate a greater “printability” of that type of mortar. Obviously, this evaluation must be associated with a verification of the “buildability”. To understand what has been said, take the case of the RMA100 mix: observing the variability of the areas, this is among the most suitable for the production of 3DPC. However, examining the elements produced, as specified previously, it is possible to deduce a poor ability to support the subsequent layers (low buildability).

This discrepancy is due to the fact that the simple value of the measured area does not take into account the irregularity of the shape of the layers following printing. Consequently, mixtures that have a lower area variability may present a more accentuated variation in shape. For this reason, thanks to the measurements carried out on the individual layers (described in Section 3.1), a shape factor, aspect ratio $A_{R}$, was defined for each of them through the following formula:

$$A_{R}=\frac{d_{min}}{d_{max}}$$

where

- $d_{min}$ is the minimum dimension, equal to the height of each layer;
- $d_{max}$ id the maximum dimension, equal to the width of each layer.

**Figure 18:**
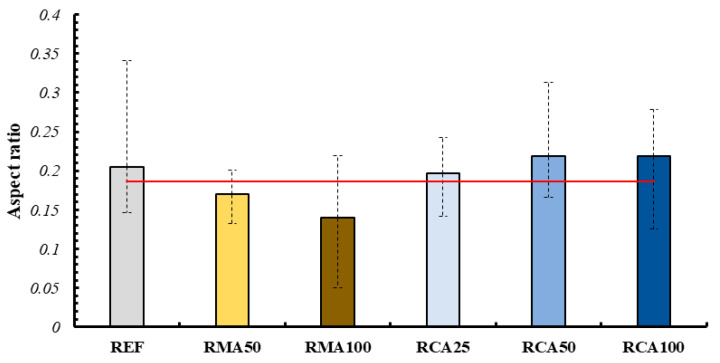
Mean aspect ratio of each layer for each mixture (red line representing the aspect ratio of the nozzle).

The results of the analysis are shown in Figure 18. This value was compared with the aspect ratio of the nozzle. As can be seen, through this analysis it was possible to highlight what was observed during the creation of the samples: more fluid mortars corresponded to a lower shape factor and therefore a greater variation from the reference constituted by the nozzle.

With this in mind, the RCA25 and RCA100 mixes are among the most suitable for 3DPC creation. The possibility of improving the other mixtures by acting on the quantities of their constituents is understood.

### 3.5. Flexural and Compressive Strength

#### 3.5.1. Compressive Strength

**Figure 19:**
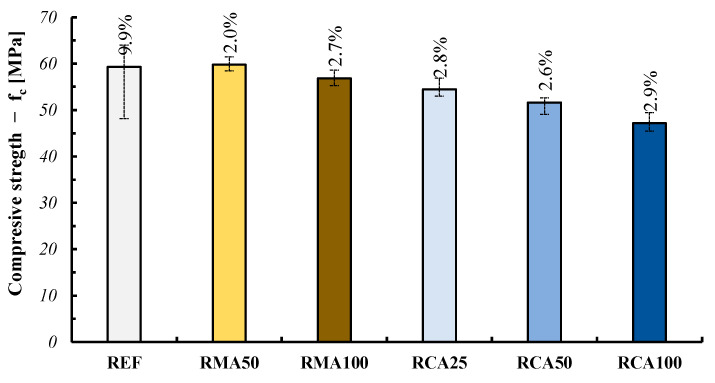
Compressive strength results on casted samples (including CoV).

Figure 19 summarizes the compressive strength results performed on the cast samples of the produced mixtures.

The results highlight that the mixture proportioning method adopted for the recycled concrete guaranteed that the required strength was reached and, moreover, no relevant difference was registered in comparison with the natural REF mix: this is more evident for the RMA mixtures; meanwhile, the RCA ones registered some small decay of their compressive strength. The higher scatter obtained for the REF mix can be associated with the lower workability of the produced natural mixtures in comparison with the recycled ones.

#### 3.5.2. Flexural Strength

**Figure 20:**
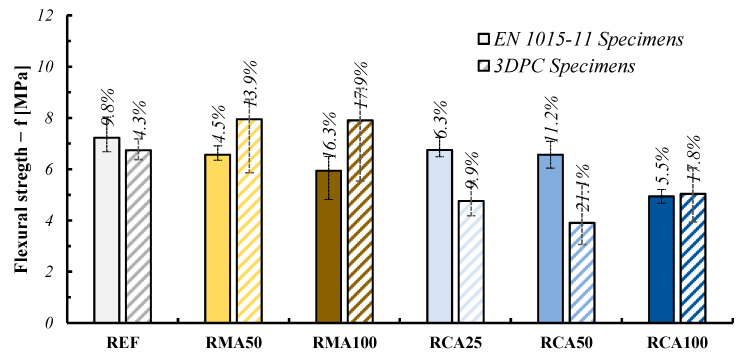
Flexural strength results—comparison between cast and 3DPC specimens (including CoV).

In line with the compressive strength results, the flexural strength performances confirmed the adequate strength of the recycled mixtures as well as the trend observed for the compressive strength; in this case, the comparison between the cast and printed samples is presented in Figure 20.

**Figure 21:**
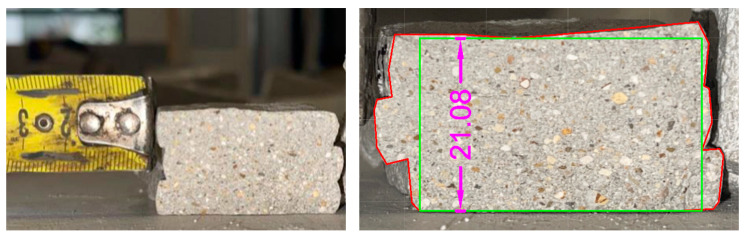
Flexural strength results—comparison between normal and 3DPC specimens.

Several studies in the literature [29,30] remark that it is possible to note that the compressive and flexural strength for printed elements in the parallel direction to the individual mortar layers is comparable to what can be generally obtained for a mortar prism made in a “single casting”. On the other hand, Figure 21 shows the red line representing the contour of the transverse cross section (as described in Section 3.4) for the extruded elements which was considered for the evaluation of their flexural strength.

The evidence that the flexural strength results of the cast and printed samples were comparable within each only in the case of the REF mixture indicates that the printed layers can present relevant defects and/or weakness points for the produced samples. As a matter of principle, the anisotropic behavior registered for the RMA and RCA mixtures can be directly associated with the shape retention and stability already discussed in Section 3.1 (see Figure 14 and Figure 15), which follows the same trend of the scatter registered in Figure 20 between the cast and extruded samples. As a matter of fact, other research in the literature [31] points out that micro-porosity is always present and measurable between the layers, which can affect the mechanical behavior of the element. However, this aspect is likely to be more apparent and measurable as 3DPC production is scaled up towards real-world structural members.

It is useful to specify that the results obtained are following an initial phase of realization of the mixes with recycled aggregate. This entails the possibility of improving the results obtained by refining the composition of the mixes. In fact, during the manufacturing phase, some promising samples were obtained with encouraging mechanical characteristics but with a variability of shape; furthermore, this shape was different between the first and last elements made. This is probably due to the “unsophisticated” casting method and the variation of workability over time, a parameter that is certainly fundamental for 3DPC and which therefore needs to be explored further.

## 4. Conclusions

In this study, the main physical and mechanical parameters characterizing various cement mortar mixtures were analyzed. These mixtures were differentiated from each other according to the type of fine aggregate used as inert material: in particular, three types of aggregate were used, two of which came from the recycling process of concrete and bricks, respectively, and were used in volume percentages that varied between them.

The values of consistency and resistance to compression and flexure were then obtained from these mixtures. Furthermore, elements were produced by simulating the 3D printing process of concrete in order to observe whether these mixtures were suitable for this purpose. The elements thus obtained were also subjected to tests to evaluate their resistance to flexure, so as to be able to compare them with the reference samples.

From the analysis of the results obtained, it was possible to observe first of all how the use of even high percentages of recycled aggregates does not imply an impossibility in the use of mortars as a suitable material for the creation of structural 3DPC elements. The mechanical performances obtained, in fact, are comparable and in line with this use.

Regarding the future development of this research, it is considered necessary to improve and standardize the process of producing 3D extruded elements, possibly using a specific machine. It will then be possible to proceed with the investigation of other aspects characterizing the various mixtures, also deepening the study of aspect related to the anisotropic nature of 3DPC.

The main limitations regarding the tests carried out are identified mainly in the operational difficulty of producing standardized specimens: the use of a manual hopper, in fact, involves variability that is not easily controllable in the production phase, mainly linked to factors arising from production by an operator rather than by machinery, such as the variable speed of creating the layer of mortar and the variable pressure applied during the extrusion phase (calibrated manually with a screwdriver).

Possible adjustments and solutions to apply to the mechanism used could include the following:

- Blocking the manual hopper in plane so that it acts as a “mortar pump” that is easier to purchase and use;
- The use of a tube, for example in plastic material, to be attached to the manual hopper and used as an “arm” that is easier to manage by the operator;
- The creation of a special frame in which to place the nozzle, in a vertical position, which can move in space and therefore is able to simulate the movement of the printing machinery more effectively. All this is considered with a view to a more economical and accessible simulation of the 3D printing process.

As regards the mechanical characteristics of the 3DPC elements, a larger campaign is needed, in which flexural and compression tests in the three main directions have to be executed, to be able to highlight any anisotropic behavior of the materials. This first phase, in fact, was helpful to understand whether or not it was possible to use recycled aggregates as effective substitutes for conventional aggregates and how their quantity affected some fundamental parameters of the mortar in the context of 3D concrete printing. Finally, it was also possible to evaluate the effectiveness of the method used for the production of 3DPC, which is considered promising as a possible simulation of conventional printing machines, which are more difficult to access.

A further limitation of the proposed study concerns the anisotropic behavior of the 3DPC elements in their hardened state. In fact, as is known, these present a different mechanical response depending on the direction in which the stress is applied. From this point of view, a modeling that could be applied, given the small dimensions of the elements produced, could be based on interface-based approaches [11,15]: starting from the assumption that such mechanical behavior is linked to the sole presence of the horizontal and vertical interface surfaces between the various layers, the laws describing the bond at the interface could be experimentally developed and the individual layers could be modeled as homogeneous continua. Once this is done, such models could be validated with a further experimental campaign.

## Figures and Tables

**Table 1:** Composition of the mixtures under consideration.

Mix Code	Vol	Cement	W_tot_	SP	f_NAT_	f_RCA_	f_RMA_
-	m^3^	kg/m^3^	kg/m^3^	kg/m^3^	kg/m^3^	kg/m^3^	kg/m^3^
REF	0.002	800.00	297.03	2.50	1135.61	0.00	0.00
RMA50	0.002	800.00	400.00	5.00	567.80	488.31	0.00
RMA100	0.002	800.00	392.13	5.00	0.00	0.00	849.43
RCA25	0.002	800.00	320.00	2.50	850.00	190.00	0.00
RCA50	0.002	800.00	340.00	3.75	567.80	385.00	0.00
RCA100	0.002	800.00	380.00	5.00	0.00	770.00	0.00
REF	0.002	800.00	297.03	2.50	1135.61	0.00	0.00

## Data Availability

The original contributions presented in this study are included in the article. Further inquiries can be directed to the corresponding author.
